# Assessing Exposures to Magnetic Resonance Imaging’s Complex Mixture of Magnetic Fields for *In Vivo, In Vitro*, and Epidemiologic Studies of Health Effects for Staff and Patients

**DOI:** 10.3389/fpubh.2018.00066

**Published:** 2018-03-12

**Authors:** Jennifer Frankel, Jonna Wilén, Kjell Hansson Mild

**Affiliations:** ^1^Department of Radiation Sciences, Radiation Physics, Umeå University, Umeå, Sweden

**Keywords:** electromagnetic field, occupational exposure, switched gradient field, diagnostic imaging, measurement

## Abstract

A complex mixture of electromagnetic fields is used in magnetic resonance imaging (MRI): static, low-frequency, and radio frequency magnetic fields. Commonly, the static magnetic field ranges from one to three Tesla. The low-frequency field can reach several millitesla and with a time derivative of the order of some Tesla per second. The radiofrequency (RF) field has a magnitude in the microtesla range giving rise to specific absorption rate values of a few Watts per kilogram. Very little attention has been paid to the case where there is a combined exposure to several different fields at the same time. Some studies have shown genotoxic effects in cells after exposure to an MRI scan while others have not demonstrated any effects. A typical MRI exam includes muliple imaging sequences of varying length and intensity, to produce different types of images. Each sequence is designed with a particular purpose in mind, so one sequence can, for example, be optimized for clearly showing fat water contrast, while another is optimized for high-resolution detail. It is of the utmost importance that future experimental studies give a thorough description of the exposure they are using, and not just a statement such as “An ordinary MRI sequence was used.” Even if the sequence is specified, it can differ substantially between manufacturers on, e.g., RF pulse height, width, and duty cycle. In the latest SCENIHR opinion, it is stated that there is very little information regarding the health effects of occupational exposure to MRI fields, and long-term prospective or retrospective cohort studies on workers are recommended as a high priority. They also state that MRI is increasingly used in pediatric diagnostic imaging, and a cohort study into the effects of MRI exposure on children is recommended as a high priority. For the exposure assessment in epidemiological studies, there is a clear difference between patients and staff and further work is needed on this. Studies that explore the possible differences between MRI scan sequences and compare them in terms of exposure level are warranted.

## Introduction

Potential risks from exposure to electromagnetic fields (EMF) have been investigated for many years. Today there is a consensus about the effects of exposure to strong fields, and international organizations such as ICNIRP (1–3) and IEEE (4, 5) have issued guidelines for the maximum allowed occupational exposures. In 2013, the EU (6) issued a directive on occupational EMF exposure which has been implemented as national standard in the member states.

How weak fields could affect human health is still a question under debate. If the EMF exposure is below the national standard limits, there are, according to our currently available knowledge, no risks associated with the exposure, either in the long or short term. However, research has shown that fields can exert an effect on biological systems below these limit values, but it is not known how this takes place, i.e., the interaction mechanism is not known, and it is not known if these effects are hazardous to our health, see further SCENIHR (7). The WHO expert agency for research on cancer, IARC, has classified both low-frequency magnetic fields (8) and radiofrequency (RF) fields (9) as possibly carcinogenic to humans, class IIB.

Very little attention has been paid to the case where there is a combined exposure to several different fields at the same time. This is the case in magnetic resonance imaging (MRI), which we will look more closely at here.

Magnetic resonance imaging is a technique that has a huge significance for today’s diagnostic activities in health care, with high resolution, good soft tissue contrast, and a lot of flexibility regarding what types of tissue properties can be featured.

In Sweden, today we have an estimated 150 MRI scanners, and there are approximately half a million MRI exams performed each year. Worldwide more than 150 million examinations have been carried out in total as estimated by Bonello and Sammut (10). Therefore, a large number of people are exposed to the EMF associated with MRI scans. In addition to this, staff who control the scanners are exposed, in different amounts depending on their role during the examinations.

In MRI, a very complex mixture of EM fields is used: static, low-frequency, and RF magnetic fields. The static magnetic field (SMF) ranges from one to several Tesla (T) for different scanners. The low-frequency field is turned on and off in various patterns, and the peak flux density can reach several millitesla and with a time derivative of the order of several Tesla per second. The RF field has a magnitude of up to several microtesla giving rise to whole-body (wb) specific absorption rate (SAR) values of a few Watts per kilogram. The exposure of the patient is regulated by the CENELEC guidelines (11) and occupational exposure by an EU directive (6) which will be discussed in more detail later.

While the acute effects of EMF exposure are well understood and regulated, there is little knowledge about other possible effects such as chronic effects. Some studies have demonstrated genotoxic effects in cells after exposure to an MRI scan (12–14), while others could not demonstrate any effects (15, 16). The MRI sequences that were used are clinically available and routinely used in heart and brain scans. Foster et al. (17) criticized these studies because many lacked a positive control, sham exposure, and blinding in the analysis work. They suggested that the results should be confirmed by studies using the same endpoints but with higher statistical power and a more rigorous design. In Reddig et al. (18), patients undergoing clinical computed tomography (CT) scans were used as positive controls, and the authors found nearly a doubling of DNA double-strand breaks 5–30 min after the CT scan as compared with before the CT scan. There was no evidence of DNA damage after the MRI examinations.

Studies from Utrecht University performed risk assessments for MRI workers and found an association between MRI-related occupational SMF exposure and an increased risk of accidents leading to injury and commute-related (near) accidents during the commute from home to work (19, 20). Huss et al. (21) found that radiographers using intrauterine devices (IUDs) while they were occupationally exposed to stray fields from MRI scanners reported abnormal uterine bleeding more often than their coworkers without IUDs or non-exposed colleagues with IUDs. In particular, radiographers present inside the scanner room during image acquisition showed an increased risk. These findings point to the need for further research to find out if staff working close to MRI scanners are at increased health risk.

Hansson Mild et al. (22) pointed out the need for well-designed epidemiological studies of MRI workers. Due to the complex mixture of SMFs, switched gradient magnetic fields, and RF EMFs, it is necessary to discuss how to evaluate the exposure in epidemiological studies. Frankel et al. (23) started developing a method for assessing the magnetic field exposure of the MRI patient during different MRI protocols so that a useful exposure metric will be available for future epidemiological studies.

The EMFs associated with MRI scanners have been studied closely, for example by Capstick et al. (24), and have been discussed at length by McRobbie (25). Therefore, only a summary is given here. In addition to the strong SMF (currently usually 1.5 or 3 T), every MRI scanner requires a switched gradient field and a pulsed RF field.

During recent years, integrated PET/MRI imaging, which combines full MRI and positron emission tomography, has been introduced. However, there are still many questions about when to use PET/MRI scanning, what radiopharmaceutical to use, and how to optimize examination protocols. It is beyond the scope of this paper to go deeper into this, and the reader is referred to Ferda (26) for further information on this topic, but from an exposure point of view, the magnetic fields from the MRI scanner are combined with exposure to ionizing radiation from the radiopharmaceuticals.

Our primary focus here will be to describe the complex mixture and combination of various fields, with intermittency and different duty cycles for the RF field, the varying shape and magnitude of the gradient field exposure, and the SMF strength. We also wish to highlight other types of exposure that could be present at the same time, especially for the patient.

## Static Magnetic Field

The SMF is always on regardless of whether the scanner is active or not. The flux density used in MRI scanners is typically 1.5 or 3 T, and now even 7-T machines are available, mainly for research purposes. This leads to exposure of the staff as well as the patients. Batistatou et al. (27) measured the exposure to SMF and motion-induced time-varying magnetic fields (TVMFs) in MRI staff in clinical practice in the UK. In total, 98 individuals, mainly radiographers, participated in the study. The average geometric mean peak SMF and TVMF exposures were 448 mT (range 20–2,891) and 1,083 mT/s (9–12,355 mT/s). The time-weighted exposure to SMF was found to be 16 mT (range 5–64). Recently, Andreuccetti et al. (28) measured the exposure of operators moving in the static field, and they found that the time derivative d*B*/d*t* could reach several Tesla per second.

It is known that strong SMFs can cause unpleasant sensations. These have been associated with induced electric fields in the body due to movement in the SMF. Several studies have investigated subjective symptoms experienced by the staff when moving in the scanner room. Zanotti et al. (29) found that the main symptoms were: unusual drowsiness/tiredness, concentration problems, headaches, sleep disorders, nausea, illusion of movement, and dizziness/vertigo. Wilén and de Vocht (30) found that reporting of health complaints was related to the strength of the magnet(s) where the nurses worked, and 57% of the symptoms were reported by the nurses who worked with the strongest systems—both 1.5- and 3-T scanners. Schaap et al. (31) found that vertigo among people working around MRI scanners was associated with motion-induced TVMFs. As a precaution, medical personnel should move slowly within the field gradient, see further ICNIRP (32, 33). For the patients, this is only a problem when entering the magnet and its steep gradient in the static field. Well inside the magnet, the static field is designed to be constant.

It is well known that particular attention must be given to ferrous material in the scanner room. The magnet attracts such objects, and they become dangerous projectiles. There is the risk of hitting the patient, operators, and the equipment in the room. Typical objects at risk are oxygen and helium cylinders, IV stands, cleaning trolleys, chairs, lamp holders, scissors, forceps, clampers, traction weights, monitoring instruments, and especially metallic splinters within the patient. The effect on implantable medical devices also needs to be taken into account. These safety hazards exist only when there are failures in the implementation of routine safety measures.

## Low-Frequency Switched Gradient Field

**Figure 1:**
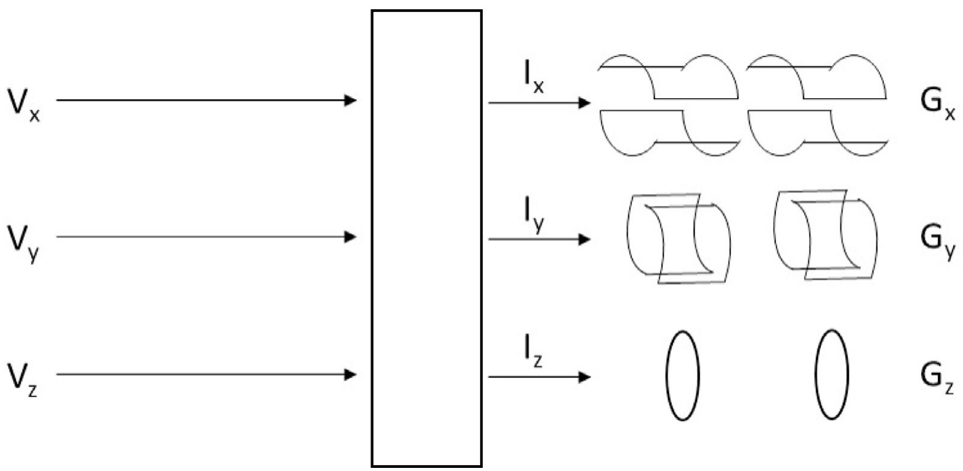
Illustration of the three gradient coils where a voltage on each of the three coils (*x*,*y*,*z*) produces current (*I*) in each coil, which results in spatial gradients (*G*) in millitesla per meter.

The gradient field provides temporary gradients in the static field along the scanner’s three axes and is produced by three large coils, one for each axis; for further details, see Figure 1. The gradient field is necessary for obtaining the spatial allocation of the individual MR signals reflecting the anatomical structure. The nuclear spins will show different precessional frequencies at different positions.

As the gradients are switched on and off the resulting gradient field varies at frequencies in the hertz to kilohertz range. The gradient field is 0 at the isocenter and reaches a maximum near the edges of the coils with an intensity of several mT_rms_. Given the short rise and fall times (tens to hundreds of microseconds), the time derivative can be substantial, and there is a need for a limit value in order not to reach the threshold of nerve excitation which would be in the order of hundreds of Volts per meter.

**Figure 2:**
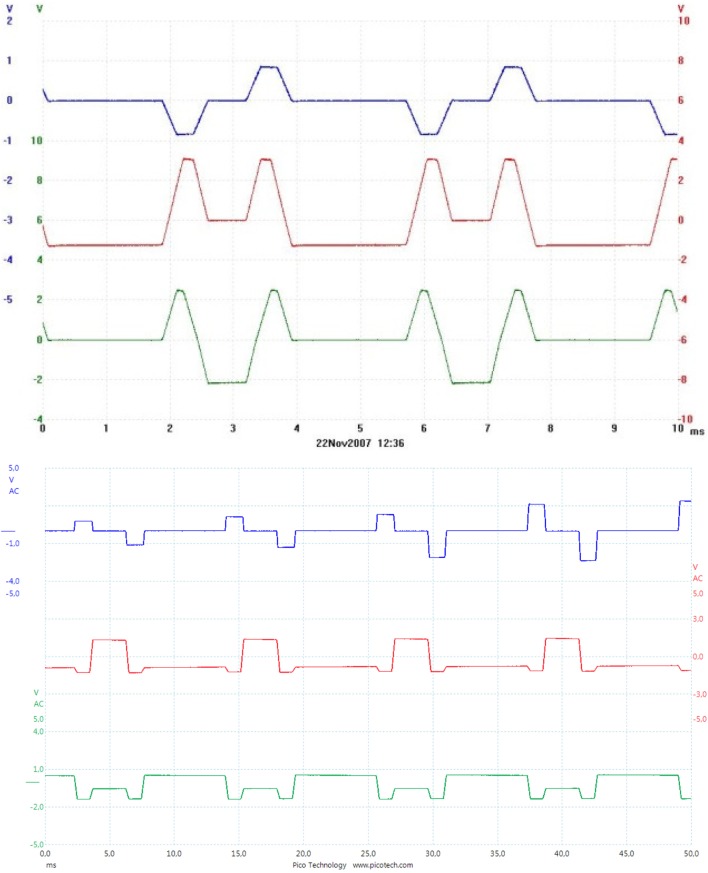
Example of the current in the three gradient coils in a Trufi sequence (above) and a T_2_-TSE sequence (below) on a Siemens Espree 1.5 T. Scale 1 V/100 A. The three coils are labeled X = blue, Y = red, Z = green.

**Figure 3:**
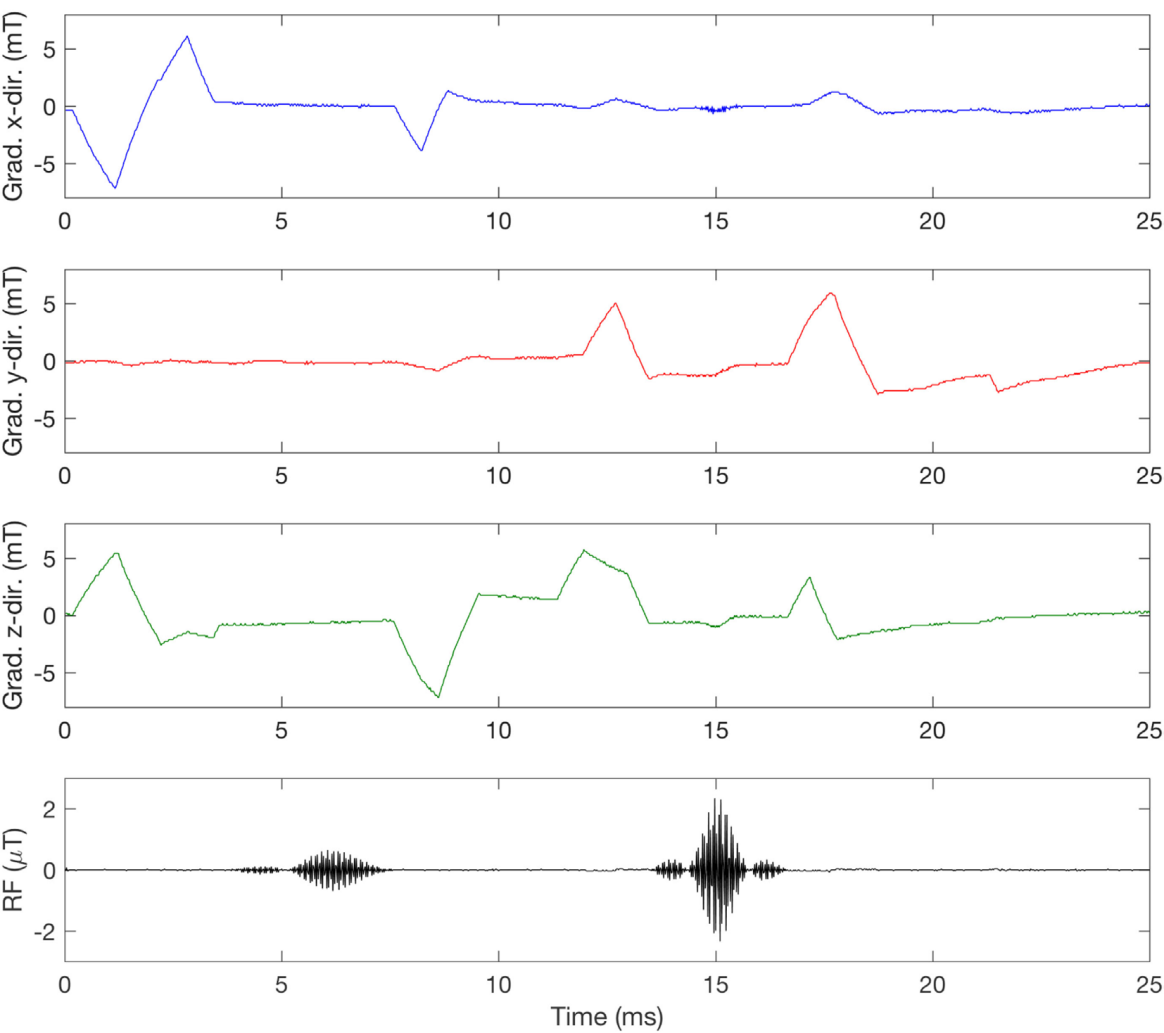
Measurement of the switched gradient magnetic field in a GE SIGNA PET/MR 3 T during a T_1_-weighted spin echo (SE) sequence. The measurement was taken at coordinates x =10, y = 13, z = 32 cm relative to the isocenter, with a Narda ELT-400 and a three-dimensional probe aligned with the scanner coils so that X is blue, Y is red, and Z is green. The radiofrequency (RF) pulses were picked up by a single-axis coil, showing the typical 90° and 180° pulses in the SE sequence.

Figure 2 shows the current being sent through each of the three gradient coils for two types of sequences, in this case producing gradients in all three directions simultaneously. In contrast to this smooth signal, when the gradient magnetic field is measured inside an MRI scanner bore, the signal may look a bit noisy. This is partly due to concomitant fields, but can also result from, e.g., amplifier noise (34), and of course measurement errors. Figure 3 shows the *x*-, *y*-, and *z*-directed gradient fields, measured inside the bore of a GE 3-T scanner, about 36 cm from the isocenter, where the gradient field is not 0. Depending on which sequence is used, the magnetic field and the d*B*/d*t* will vary, as is indicated by the differences in the current for the two sequences in Figure 2. This has also been shown by Wilén et al. (35).

## RF Field

The RF field consists of short pulses at specified intervals, and its carrier frequency is directly related to the strength of the static field according to the gyromagnetic ratio for protons of 42.58 MHz/T. Therefore, on a 1.5-T scanner, the RF frequency is 63.87 MHz, and on a 3-T scanner, it is 127.74 MHz. The RF field is usually circularly polarized.

Specific absorption rate values are limited by the scanner in accordance with CENELEC regulations (11) to prevent the patient from overheating from the RF field. The scanner mode defines the SAR limit, and there are three different levels to choose from. In *normal operating mode*, the default wb SAR should be kept below 2.0 W/kg over any 6-min period. In *first-level controlled operating mode*, SAR is allowed to reach 4.0 W/kg. These two modes are used clinically. *Second-level controlled operating mode*, however, allows for SAR values >4.0 W/kg, but is mainly used in research, and requires explicit ethical approval. The choice of operating mode will also affect the maximum allowed d*B*/d*t* level.

**Figure 4:**
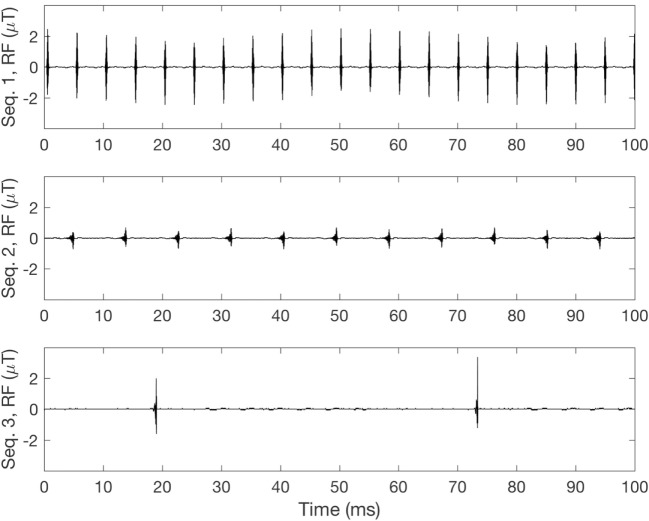
Measurement of radiofrequency (RF) pulses for different sequences. Sequence 1 (top): 8% duty cycle, SAR_wb_: 1.05 W/kg. Sequence 2 (middle): 9% duty cycle, SAR_wb_: 0.07 W/kg. Sequence 3 (bottom): 2% duty cycle, SAR_wb_: 0.06 W/kg.

The two modes used clinically ensure that the average RF exposure does not exceed the given limits. However, since the RF field is pulsed, and sometimes with a low duty cycle, the peak exposure can be high. As an example, for a sequence with an estimated wb SAR (6 min) of 1 W/kg and a duty cycle of 1%, SAR during each pulse would reach 100 W/kg. This type of exposure has been studied very little so far. Also, the RF field will vary depending on the sequence used (23) and an example of this difference can be seen in Figure 4.

## MRI Exams

A typical MRI exam includes several imaging sequences of varying length and intensity, to produce several different types of images. Each sequence is designed with a particular purpose in mind, so one sequence can, for example, be optimized for clearly showing fat water contrast, while another is optimized for high-resolution detail. Since each additional sequence adds to the total time the patient has to spend in the scanner, the sequences included in an exam need to be chosen carefully to provide a maximum amount of relevant information, without the exam going on for too long.

Table 1 shows an example of an MRI head exam with a total scan time of approximately 21 min. The sequences^1^ included in this exam vary in length between 20 s and 7 min and have varying levels of SAR.

**Table 1:** Sequence list from an MRI head scan protocol run in the first level controlled operating mode on a GE 3-T scanner.

Sequence	Scan time (mm:ss.ms)	Field of view (mm)	Slice thickness (mm)	Flip angle (°)	SAR_6 min_ (W/kg)
3Plane Loc SSFSE*	00:19.87	300	10	90	3.199
Ax FSPGR 3D	2:06.80	200	1	12	0.140
Ax diffusion-weighted image	2:37.60	220	3.6	90	0.294
Sag CUBE FLAIR	6:57.16	240	1.2	90	0.363
Ax FSPGR 3D	2:04.71	200	1	12	0.140
Ax T2 PROPELLER	6:46.30	220	2	142	2.548
Total time	20:52.44				

The sequence denoted * is a localizer, described below. The SAR_6 min_ is a whole-body average value.

Every exam starts with a quick localizer sequence that produces a low-resolution image used for planning. The scanner technician uses this image to determine the slice positions.

In this example, the next sequence is an FSPGR, a gradient echo sequence which produces a three-dimensional image slab rather than a slice. The imaged volume can then be studied from any direction, by placing “digital slices” on any axis.

The diffusion-weighted image is useful for detecting and differentiating areas of subtle histological variation, for example, lesions, cysts, tumors, and areas of ischemic stroke.

The CUBE FLAIR sequence suppresses the MR signal from fluids, allowing contrast between other tissues to become more visible.

The FSPGR 3D sequence was included twice in this exam because the images from the first round did not come out exactly as desired. With some minor adjustments to the setup and instructions to the patient to try to be perfectly still, the sequence was re-run with more satisfying results. In some cases, a sequence will be included twice in an exam to re-do unsatisfactory images (e.g., due to patient movement), while in other protocols, a sequence is scheduled to be run twice to generate comparable images before and after contrast solution has been administered to the patient.

The final sequence in Table 1, PROPELLER, uses a special motion correction technique, which is helpful to reduce image artifacts due to voluntary or physiologic patient motion.

Prefixes such as “Ax” and “Sag” refer to the direction of the image slices and slabs, describing whether they are placed in the axial (Ax), sagittal (Sag), or coronal (Cor) plane.

As can be seen in Table 1, the sequences included in this exam differ in duration and SAR, and in many other sequence-specific parameters as well, such as field of view (FOV), slice thickness, and flip angle, etc. The different sequences in an exam produce different kinds of images, all contributing to the set of information that will then be available to the radiographer for analysis.

### Contrast Agents

It is very common to use contrast media in MRI. Lin and Brown (36) estimated that nearly 40–50% of all scans were done with the use of a contrast medium. The most common one is Gadolinium (Gd), a paramagnetic substance and a rare earth metal, which is toxic in its free state. Therefore, when used, it is bound to other chemicals by chelation. The contrast medium affects both *T*_1_ and *T*_2_ relaxation times. Contrast media are not distributed evenly throughout the body and signals from different tissues will be influenced differently, which can be helpful when differentiating between tumor tissue and surrounding edema.

One medium often used is Dotarem, which is based on Gd. The dosage is normally 0.2 ml/kg body weight. Other examples are Magnevist and Primovist. Typical side effects reported are rather mild with Dotarem (urticaria and nausea), but other types of contrast media may cause an increased risk of nephrogenic systemic fibrosis, see further Thomsen et al. (37).

To our knowledge, no studies have been done to further explore the role of combinations of EMF exposure with these chemical agents.

### Ionizing Radiation

During PET/MR procedures, the patient will also be exposed to ionizing radiation from the administered radiopharmaceutical. The effective dose to the patient will vary significantly depending on the radiopharmaceutical used, the weight of the patient, and other factors as well (38). A separate exposure assessment of the ionizing radiation needs to be performed if PET/MRI patients are included in, for instance, epidemiological studies.

## Exposure and Dose

To determine if there is a possible health risk associated with exposure to EMF, it is necessary to know what constitutes “dose” in this context. Present exposure guidelines (1, 3, 6) are focused on eliminating acute thermal effects from high-frequency (RF) field exposure, sensory effects such as dizziness, vertigo, etc. from movement in SMFs, and neuroexcitation from low-frequency field exposure. The guidelines for RF exposure are given as the SAR (Watts per kilogram), a dose rate metric which is proportional to the 6-min average of the square of the induced electric field (*E*^2^). For low-frequency fields, induced E-field in the tissue is used, which is proportional to the external rate of change of the switched gradient magnetic field. Both of these variables are dose-rate measures that give a real-time image of how the electric field is distributed within the tissue. In studies where other biological end points than heating, sensory effects and neuroexcitation are of interest, it may be too simplistic to equal the dose taken up by a biological structure (cell, tissue, organ, organism) with the strength of the electric field multiplied by the exposure time. We argue that other factors may need to be considered as well, to end up with a dose measure that is biologically relevant for the specific endpoints being studied. So far, this discussion has not really been pursued. Hypotheses on what constitutes exposure and dose are not clearly defined or even discussed in EMF research, primarily because the interaction mechanism(s) are not well understood, especially concerning weak fields and non-thermal effects. Hansson Mild and Mattsson (39) argue that there is a need to look at different meanings of the dose concept with regard to different symptoms and diseases, and also different organ/tissue sensitivities.

In the MRI environment, where strong static, switched gradient, and RF magnetic fields are applied, the induced E-fields of all these three field types must be limited to ensure that patients and staff do not experience acute health effects. In research, where other biological or health related endpoints are studied, we argue that other exposure parameters could be relevant. The switched gradient magnetic field, for instance, changes rapidly, and depending on what sequence is used and the slew rate of the gradient coils, the *B*, d*B*/d*t*, and d*B*/d*t*(max) can all vary. The same is true for the RF field where the number of RF pulses per second varies depending on the sequence used. Also, certain sequence settings, such as flip angle, can impact the amplitude of the RF pulse, as has been shown by Frankel et al. (23).

Different theories exist about the interaction mechanisms of weak magnetic fields; for example, Litovitz et al. (40) showed that in order for an EMF exposure to cause a biological effect the signal must be both coherent and constant over a time period of at least 10 s. If we apply Litovitz’s theory to possible biological effects of the switched gradient field in the MRI scanner, the coherence criteria are not fulfilled. The amplitude and direction of the gradient field change constantly, as the currents in the three coils change; see for instance Figures 2 and 3. Other interaction mechanism(s) that are being discussed include the radical pair mechanism, see further Ref. (41), in which reactions are strongest when static MFs are in resonance with either ELF or RF magnetic fields. To what extent this can occur in MRI is not clear.

## Discussion

Exposure metrics suitable for acute effects, such as SAR for heating and induced E-fields for peripheral nerve stimulation, are perhaps not appropriate metrics when studying other effects and low-level exposures. For combined exposures in the MRI environment, exposure assessment is even more challenging since multiple fields are present at the same time, perhaps together with contrast media chemicals such as Gadolinium, and in some cases with ionizing radiation from PET/MRI scanning. This is relevant for *in vitro, in vivo* as well as for epidemiological studies.

### *In Vivo* and *In Vitro* Experiments

In view of what has been shown here about the complexity and mixture of different EMFs in connection with an MRI examination, it becomes rather apparent that previous *in vivo* and *in vitro* studies will be almost impossible to replicate due to the lack of information about exposure and dose. Let us take a closer look at some of these studies.

The study by Simi et al. (14) involved both *in vitro* and *in vivo* experiments. Regarding the exposure we are told what scanner was used and its slew rate. The exposure consisted of five different sequences: gradient echo, steady-state free precession, triple inversion recovery, FastSE, and EPI-derived perfusion. For the *in vitro* experiment, we are not told where in the scanner the samples were placed: isocenter or elsewhere?

Lee et al. (13) investigated the genotoxic potential of 3-T clinical MRI scans on cultured human lymphocytes *in vitro*. The cells, held in T-25 flasks, were placed in the isocenter, a clinical routine brain protocol was applied (three-channel head coil), and the MRI scan sequence protocol included six different pulse sequences (Axl T2-FSE, Axl T2-FLAIR, Axl T1-SE, Axl DTI, Sag T1-FLAIR, Cor T2-FSE).

Fiechter et al. (12) looked at blood samples from twenty consecutive patients referred for cardiac evaluation. In a Philips 1.5-T Achieva instrument they applied standard pulse sequences to generate images: gradient echo, steady-state free precession, FastSE, T2-weighted double-inversion black-blood spin echo sequence for edema imaging, balanced SSFP sequence for perfusion, and inversion recovery segmented gradient echo sequence for late gadolinium enhancement.

The *in vivo* study by Reddig et al. (18) looked at DNA double-strand breaks in lymphocytes from patients scheduled for an MRI exam in 1-, 1.5-, or 3-T machines. In some cases, contrast agents were used. The only thing reported regarding exposure was the estimated total amount of absorbed energy according to each of the different MRI protocols used. The standardized energy dose (SED = mean whole-body SAR × exposure time) ranged from a low 182 to a high 2,825 J/kg.

Recently, Sannino et al. (42) looked at mitomycin C-induced chromosomal fragility in peripheral lymphocytes from 12 MRI workers whose exposure was limited to movement in the SMF. They found a high worker-to-worker variability, but no specific exposure assessment was done with regard to these effects.

Another approach has been taken by Wilén et al. (43) where a dedicated exposure system was developed to expose lymphocytes to RF pulses similar to those found in clinically used MRI protocols. The exposure system was placed inside a cell culture incubator with a weak ELF background field (44), with the advantage that exposure, temperature, and humidity were well controlled and adjustable. This experiment was performed on the assumption that the RF pulses are what cause genotoxic effects, and not the low-frequency magnetic field or the SMF.

It is of the utmost importance that future experimental studies give a thorough description of the exposure they are using, and not just a statement such as “An ordinary MRI sequence was used.” Even if the sequence is specified, it can differ substantially between manufacturers on, e.g., RF pulse height, width, and duty cycle. Also, dosimetric evaluation is important. The placement of test tubes, flasks, or Petri dishes with cells inside an MRI scanner is quite different from scanning humans, from a dosimetric point of view. For example, the SAR_wb_ value expressed by the scanner is not relevant for test tubes. Knuuti et al. (45) discussed the clinical significance of the DNA findings and concluded that it is evident that further larger studies are needed. It is important to give much more attention to the exposure and dosimetric evaluation in future research, either by calculation or measurement of the magnetic field strength at the position of the test tubes, or by calculation of the absorption within the test tubes.

### Epidemiology

In the latest SCENIHR opinion (7), it is stated that there is very little information regarding the health effects of occupational exposure to MRI fields, and long-term prospective or retrospective cohort studies on workers are recommended as a high priority. They also state that MRI is increasingly used in pediatric diagnostic imaging, and a cohort study into the effects of MRI exposure on children is recommended as a high priority.

For the exposure assessment in epidemiological studies, there is a clear difference between patients and staff. Most members of the staff will not be present in the scanner room during scanning and, thus, they are only exposed to the SMF and the induced field due to movement in the SMF. However, some of the staff may in some situations be present during scanning. This can, for instance, be during scanning of small children or sedated persons. The patient, on the other hand, will be exposed to all three fields, and then, depending on the sequences used, different exposures will result.

For studies on MRI workers, exposure classification has been discussed by Hansson Mild et al. (22). Since the exposure from MRI equipment is a mixture of SMFs, switched gradient magnetic fields, and RF EMFs, it is not clear how they should be combined into a classification scheme. The first step may be to use job title as a proxy for exposure, with different levels of exposure for the different professions in the MRI environment. As an alternative to that approach, Hansson Mild et al. (22) suggested an exposure categorization for professionals working with MRI equipment. Specifically, it was proposed that exposure should be defined into three categories, depending on whether people are exposed to only the static field, to the static plus switched gradient fields, or to the static plus switched gradient plus RF fields, as a basis for exposure assessment in these types of studies. More knowledge about exposure variation between different MRI worker categories is needed, which is also highlighted in Ref. (27).

How to assess the exposure of patients undergoing MRI scans is also an open question. In contrast to the staff, who move around in the scanner room making occupational exposure assessment quite challenging, patients are exposed to well-defined field levels inside the MRI scanner bore. Patient exposure depends more on the scan protocol used than on the patient’s position, at least during scanning. However, the RF and gradient magnetic fields are complex and describing them in terms of exposure is not trivial. Furthermore, since a scan protocol can contain any number of scan sequences, there is perhaps a big difference in exposure between a brain scan and a knee scan, if the sequences involved are very different from each other. If one would aim to go one step further in exposure assessment for patients in epidemiological studies than merely measuring the amount of time spent in a scanner, it would be of interest to investigate possible differences between MRI scan sequences, and compare them in terms of exposure level. Frankel et al. (23) initiated work on this topic by measuring the RF and gradient magnetic fields in an MRI scanner while varying some of the many sequence parameters that can be adjusted before a scan. Preliminary results indicated that some parameters may have a large impact on exposure levels, but further work is necessary to give a comprehensive description of MRI patient exposure, if epidemiological studies are to be undertaken and expected to give meaningful results.

## Conclusion

The MRI environment is complex due to the mixture of different magnetic fields present and the multitude of exam protocols available. The patient might also be exposed to contrast media and/or ionizing radiation (PET/MR) during the procedure, and this needs to be included in the exposure assessment as well. In epidemiological, *in vivo*, and *in vitro* studies, careful exposure assessment is essential, and a hypothesis of how to define the dose is necessary. Studies that explore the possible differences between MRI scan sequences and compare them in terms of exposure level are warranted, to provide the possibility of including an MRI exposure level factor in future epidemiological studies.

## Author Contributions

JF contributed to the design of the project, measurement and analyses and drafting the manuscript. JW and KHM contributed to the design and drafting the manuscript.

## Conflict of Interest Statement

The authors declare that the research was conducted in the absence of any commercial or financial relationships that could be construed as a potential conflict of interest.

## Footnotes

^1^Sequence names in Table 1 are GE-specific. FOV field of view.
